# Maternal Fecal Microbiota Transplantation Mitigates Hypertension in Offspring Exposed to a High-Fructose Diet

**DOI:** 10.3390/antiox14101168

**Published:** 2025-09-25

**Authors:** Chien-Ning Hsu, Chih-Yao Hou, Hong-Tai Tzeng, Kay L. H. Wu, Wei-Chia Lee, Guo-Ping Chang-Chien, Shu-Fen Lin, You-Lin Tain

**Affiliations:** 1 Department of Pharmacy, Kaohsiung Chang Gung Memorial Hospital, Kaohsiung 833, Taiwan; cnhsu@cgmh.org.tw; 2 School of Pharmacy, Kaohsiung Medical University, Kaohsiung 807, Taiwan; 3 Department of Pharmacy, Kaohsiung Municipal Ta-Tung Hospital, Kaohsiung 801, Taiwan; 4 Department of Seafood Science, National Kaohsiung University of Science and Technology, Kaohsiung 811, Taiwan; chihyaohou@nkust.edu.tw; 5 Institute for Translational Research in Biomedicine, Kaohsiung Chang Gung Memorial Hospital, Kaohsiung 833, Taiwan; htay11@cgmh.org.tw (H.-T.T.); klhwu@cgmh.org.tw (K.L.H.W.); 6 Department of Urology, Kaohsiung Chang Gung Memorial Hospital and Chang Gung University College of Medicine, Kaohsiung 833, Taiwan; dinor666@ms32.hinet.net; 7 Super Micro Mass Research and Technology Center, Cheng Shiu University, Kaohsiung 833, Taiwan; guoping@csu.edu.tw (G.-P.C.-C.); linsufan2003@csu.edu.tw (S.-F.L.); 8 Institute of Environmental Toxin and Emerging-Contaminant, Cheng Shiu University, Kaohsiung 833, Taiwan; 9 Center for Environmental Toxin and Emerging-Contaminant Research, Cheng Shiu University, Kaohsiung 833, Taiwan; 10 Department of Pediatrics, Kaohsiung Chang Gung Memorial Hospital, Kaohsiung 833, Taiwan; 11 Department of Pediatrics, Kaohsiung Municipal Ta-Tung Hospital, Kaohsiung 801, Taiwan; 12 College of Medicine, Chang Gung University, Taoyuan 333, Taiwan

**Keywords:** gut microbiota, hypertension, developmental origins of health and disease (DOHaD), short-chain fatty acids, fructose, fecal microbiota transplantation

## Abstract

Excessive maternal fructose intake contributes to the developmental programming of hypertension in offspring, partly via gut microbiota dysbiosis and oxidative stress. Fecal microbiota transplantation (FMT) may restore microbial balance and modulate short-chain fatty acid (SCFA) production. We investigated whether maternal FMT from healthy donors could prevent hypertension in offspring exposed to a high-fructose (HF) diet. Pregnant Sprague Dawley rats (n = 12) were fed normal chow (ND) or a 60% HF diet from mating to delivery. Cross-FMT was performed: HF dams received FMT from ND donors, and ND dams received FMT from HF donors. Male offspring (n = 8/group) were assigned to ND, HF, ND + HF-FMT, or HF + ND-FMT groups. Offspring of HF dams developed higher systolic blood pressure (+13 mmHg vs. ND, *p* < 0.05). Maternal FMT from ND donors reduced this elevation by ~8 mmHg (*p* < 0.05). Protective effects were accompanied by higher plasma butyrate, increased expression of SCFA receptors (GPR41, GPR43), reduced renal oxidative stress markers (8-OHdG), and distinct gut microbiota profiles. Maternal FMT generated four enterotypes in offspring, each associated with differential blood pressure outcomes. These findings suggest that maternal microbiota-targeted interventions, such as FMT, can mitigate hypertension of developmental origin by restoring gut microbial and metabolic homeostasis.

## 1. Introduction

Hypertension is a major global public health concern, currently affecting over one billion people worldwide [1]. Increasingly, evidence supports that the origins of hypertension can be traced back to early life exposures, as described by the Developmental Origins of Health and Disease (DOHaD) concept [2,3]. Among these early determinants, maternal nutrition during pregnancy and lactation plays a pivotal role in shaping long-term cardiometabolic health in the offspring [4,5].

Over the past decades, the widespread consumption of nutritive sweeteners, particularly sucrose and high-fructose corn syrup (HFCS), has paralleled the global rise in obesity, metabolic syndrome, and hypertension [6]. Both sucrose and HFCS deliver a substantial fructose load, which has been implicated in elevating blood pressure (BP) and disrupting metabolic homeostasis [7,8]. Animal studies consistently demonstrate that maternal high-fructose (HF) intake during gestation and lactation adversely affects fetal programming, predisposing offspring to hypertension in adulthood [9,10,11].

Oxidative stress and gut microbiota dysbiosis are increasingly recognized as central, interrelated mechanisms driving this phenomenon [12]. Changes to maternal and offspring microbiota that curtail short-chain fatty acid (SCFA) synthesis can undermine cellular redox balance, dysregulate blood pressure and vascular responses, and reprogram immune responses. In turn, elevated oxidative stress can compromise gut barrier integrity and further alter microbial composition, perpetuating a vicious cycle that heightens the risk of hypertension.

These insights have led to growing interest in nutritional strategies targeting the maternal microbiome to prevent adverse offspring outcomes. Interventions such as prebiotics, probiotics, postbiotics, and microbial metabolites like SCFAs are being explored for their potential to mitigate the detrimental effects of maternal HF intake [13,14]. A promising, yet underexplored, approach is fecal microbiota transplantation (FMT)—the transfer of fecal matter from a healthy donor to reestablish microbial balance in the recipient [15]. While FMT has shown efficacy in treating gut-related disorders, its application in the context of developmental programming of hypertension remains underexplored.

This study builds on our previous work examining the effects of maternal HF diets on offspring BP and renal outcomes in rats [9,13,14]. We investigated whether maternal FMT from healthy, normal diet-fed donors could prevent the programming of hypertension in male offspring exposed to maternal HF diets. We also evaluated the effect of FMT from HF-fed donors on offspring BP. Male offspring were selected to reduce variability and because prior studies [16] suggest that males may be more susceptible to hypertension. Maternal FMT offers a strategy to modulate the maternal gut microbiota, which can shape the offspring’s microbial colonization and metabolic programming during critical developmental windows. Additionally, we explored the underlying microbial and molecular mechanisms contributing to these effects. These findings provide novel insight into how maternal microbiota-targeted interventions can serve as a nutritional strategy to prevent developmental programming of cardiovascular and renal disease in offspring.

## 2. Materials and Methods

### 2.1. Animal Study Design

Female Sprague Dawley rats, never previously bred, were procured from BioLASCO Taiwan Co., Ltd. (Taipei, Taiwan). Procedures involving animals received prior approval from our hospital (IACUC approval no. 2022011001) and were performed in compliance with applicable ethical guidelines. The animals were maintained in a facility accredited by AAALAC, under regulated conditions including a 12 h light/dark cycle, and were provided ad libitum access to a standard chow diet and drinking water. Breeding was performed by overnight cohabitation of a single male and female, and the presence of a vaginal plug indicated successful mating.

Pregnant SD rats (n = 12) were fed either a standard laboratory rat chow (ND group) or a 60% high-fructose diet (HF group) from mating until delivery. One group of FMT was established using the ND group as donors and the HF group as recipients, while another group used the HF group as donors and the ND group as recipients.

### 2.2. Ex Vivo Fecal Microbiota Transplantation

Fresh fecal samples were collected from donor rats on gestational days 1, 7, and 14 and pooled into a homogenized mixture for ex vivo fecal microbiota transplantation. Approximately 100 mg of fecal pellets were aseptically collected into sterile tubes and homogenized in 1 mL of phosphate-buffered saline (PBS). The homogenate was centrifuged at 2000× *g* for 1 min to separate debris. The resulting supernatant, enriched in bacteria, was further centrifuged at 15,000× *g* for 5 min to pellet the bacterial fraction. This pellet was then resuspended in 1 mL of sterile saline to prepare the microbiota transplant [17]. Recipient rats received 1 mL of the bacterial suspension via enema once weekly during pregnancy, specifically on gestational days 1, 7, and 14.

Litters were standardized to eight offspring per dam shortly after birth. Male offspring were exclusively chosen for further experiments because they exhibit an earlier and heightened vulnerability to developing hypertension [16]. The male progeny was allocated into four groups (n = 8 per group): ND, HF, NDFMT (receiving fecal microbiota transplants from HF donors), and HFFMT (receiving fecal microbiota transplants from ND donors).

### 2.3. BP Measurements and Sample Collection

BP in the rat offspring was monitored weekly from 3 to 12 weeks of age using the Kent Scientific CODA system (Torrington, CT, USA) [14]. Fecal specimens were gathered and preserved at −20 °C for subsequent analysis. Twelve-week-old rats were put under anesthesia (ketamine 50 mg/kg and xylazine 10 mg/kg); euthanasia was completed with an IP pentobarbital overdose. Heparinized blood samples were collected and frozen for storage. The left kidney was perfused with cold PBS to clear residual blood before excision. A portion of the kidney was fixed in 10% buffered formalin for histopathological examination, while the remaining tissue was dissected into cortex and medulla, rapidly frozen in liquid nitrogen, and stored at −80 °C for subsequent analyses. Plasma and urine creatinine levels were measured to assess kidney function using an Agilent HP 1100 HPLC system (Wilmington, DE, USA).

### 2.4. Microbiota Analysis

Fresh fecal samples from offspring were promptly collected and stored at −80 °C until shipment to Biotools Co., Ltd. (Taipei, Taiwan) for microbial profiling [14]. Genomic DNA was isolated from these samples to perform 16S rRNA gene sequencing. A multiplexed SMRTbell library, designed for PacBio sequencing, was constructed by amplifying the full-length 16S rRNA gene using barcoded primers. Sequence data were processed through the QIIME2 platform, where phylogenetic relationships among amplicon sequence variants were inferred using the FastTree algorithm [18,19]. Microbial diversity within samples (alpha diversity) was assessed using the Pielou index and Shannon index. To compare microbial community composition between groups (beta diversity), Partial Least Squares Discriminant Analysis (PLSDA) was performed, supported by Analysis of Similarities (ANOSIM) for statistical validation. Moreover, linear discriminant analysis effect size (LEfSe) was applied to pinpoint bacterial taxa exhibiting significant differential abundance across conditions.

### 2.5. SCFAs and Receptors

Plasma levels of acetate, propionate, and butyrate in offspring were determined by gas chromatography–mass spectrometry (GC-MS) equipped with a flame ionization detector (Shimadzu QP2010; Kyoto, Japan), following established protocols [14]. Separation was achieved using a DB-FFAP capillary column (Agilent Technologies). Each analysis involved injecting 1 µL of sample with a split ratio set at 5:1, while maintaining the injector temperature at 240 °C. Quantification was normalized using 2-ethylbutyric acid as the internal standard.

Considering the known involvement of SCFA receptors in BP regulation via their binding to SCFAs [20], we measured the mRNA levels of these receptors using quantitative PCR (qPCR) with SYBR Green dye. Total RNA was isolated from the renal cortex tissue of each rat. We focused our gene expression analysis on four SCFA receptors. Expression levels were normalized against the housekeeping gene 18S rRNA (R18S) to ensure accuracy. Rat-specific primers for olfactory receptor 78 (Olfr78), G protein-coupled receptors 41 (GPR41), GPR109A, GPR43, and R18S were used as described in a previous study [14]. Each sample was analyzed in duplicate, and a dissociation curve analysis was performed following all qPCR reactions to confirm amplification specificity. Relative gene expression was quantified employing the comparative Ct (^ΔΔ^Ct) method.

### 2.6. Assessment of Oxidative Stress via 8-OHdG Immunohistochemical Staining

DNA is vulnerable to ROS-induced damage, producing 8-hydroxy-2′-deoxyguanosine (8-OHdG), a key biomarker of oxidative DNA injury [21]. Paraffin-embedded offspring kidney tissue was cut into 4 µm sections, dewaxed in xylene, and rehydrated through a descending ethanol series. Sections were then rinsed in PBS. Immunohistochemical detection of 8-OHdG was performed using a primary antibody specific to 8-OHdG (1:100, JaICA, Tokyo, Japan), and visualization was achieved through a polymer-based horseradish peroxidase conjugate system (BIOTnA Biotech, Taipei, Taiwan) with 3,3′-diaminobenzidine as the chromogenic substrate. Following staining, the tissue sections were briefly counterstained with hematoxylin and covered with mounting medium and coverslips. For negative controls, the primary antibody incubation step was omitted. All tissue sections were subjected to identical experimental conditions, including reagent concentrations, antibody dilutions, and incubation times. The number of 8-OHdG-positive cells in 10 randomly selected areas of the renal cortex was quantified using Ventana Image Viewer 3.2.0.

### 2.7. Statistical Analysis

Results are expressed as mean ± SEM. Differences among groups were evaluated by one-way ANOVA, with subsequent pairwise comparisons performed using Tukey’s post hoc test. Statistical significance was defined as *p* < 0.05. For the metabolomics analysis of alpha diversity, the Wilcoxon test was applied to evaluate whether species diversity differed significantly between groups. The ANOSIM was employed to assess the overall similarity of microbial communities, generating an R-value and corresponding *p*-value for group comparisons. Statistical analyses were performed to identify species with significant differences between groups, with multiple comparisons corrected using false discovery rate (FDR) and q-values < 0.05 considered significant. Additionally, LEfSe analysis was carried out using the Wilcoxon test to further identify discriminative features between groups. All analyses were performed using SPSS version 17.0.

## 3. Results

### 3.1. FMT Alters Offspring Outcomes in a Maternal HF Diet Model

Dams that received HF diet or FMT showed no effect on offspring mortality, as the mortality rate was zero across all groups. Notably, the NDFMT group exhibited a higher body weight (BW) compared to the ND group. Moreover, the kidney-to-body weight ratio (KW/BW) was higher in the HF group compared to the NDFMT and HFFMT groups, despite KW being similar among all four groups (Table 1). Consistent with previous studies [9,14], rat progeny revealed elevated systolic and diastolic blood pressures (SBP and DBP) and mean arterial pressure (MAP) due to maternal consumption of a high-fructose diet by week 12. Adult rat progeny born to ND dams that received FMT from HF donors had higher SBP and MAP compared to those in the ND group. Conversely, FMT from ND donors to HF dams mitigated the increases in SBP, DBP, and MAP induced by the maternal HF diet in the HFFMT group. Kidney function, as measured by 24 h creatinine clearance, showed no significant differences among the four offspring groups (Table 1).

**Figure 1:**
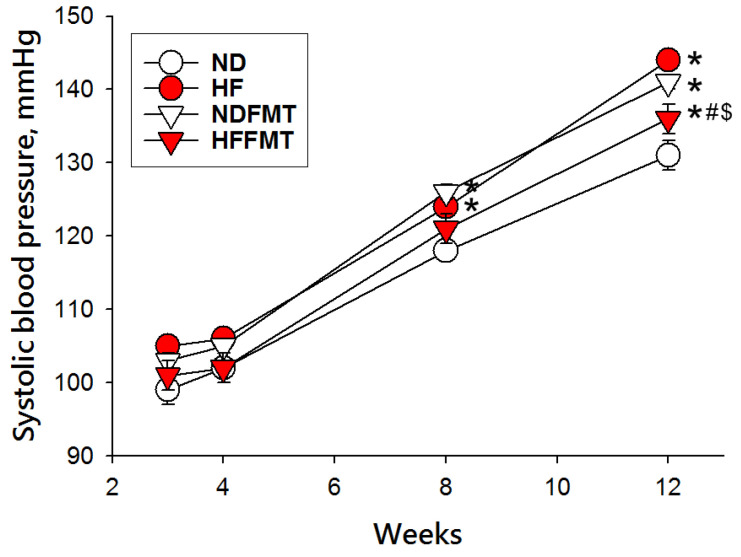
Offspring systolic blood pressure recorded from postnatal week 3 through week 12. N = 8/group. Statistical analysis by a one-way ANOVA followed by Tukey’s post hoc test. * *p* < 0.05 vs. ND; # *p* < 0.05 vs. HF; $ *p* < 0.05 vs. NDFMT.

Figure 1 illustrates the progression of SBP in rat offspring over time. Offspring born to mothers fed a HF diet exhibited a notable elevation in SBP beginning at 8 weeks of age (*p* < 0.05), which persisted through 12 weeks, relative to those in the ND group. Likewise, FMT from HF donors led to elevated SBP in the NDFMT group at both 8 and 12 weeks, relative to the ND group. However, this rise was significantly attenuated with FMT from healthy donors at 12 weeks (*p* < 0.05).

### 3.2. Maternal FMT Alters Offspring Gut Microbiota

**Figure 2:**
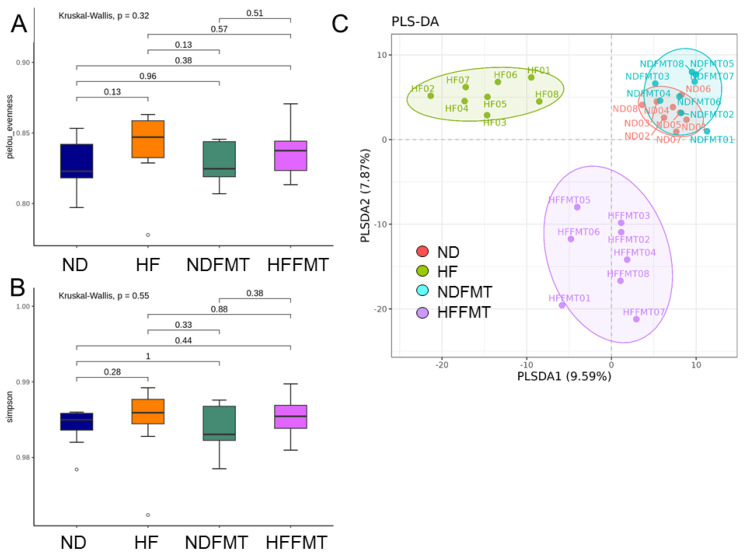
Assessment of microbial diversity among the four offspring groups included α-diversity and β-diversity analyses. (**A**) Pielou’s index and (**B**) Simpson’s index quantified species evenness and richness, respectively. For β-diversity visualization, (**C**) Partial Least Squares Discriminant Analysis (PLSDA) was performed, where each point corresponds to an individual sample’s microbiota profile, with colors indicating group membership. N = 8/group. Statistical analysis by a Kruskal–Wallis test followed by Dunn’s post hoc test.

Two α-diversity indices—Pielou’s evenness (Figure 2A) and Simpson’s diversity (Figure 2B)—were evaluated to measure species distribution and abundance. No significant differences were observed across the four offspring groups for either the Pielou index (*p* = 0.32) or Simpson index (*p* = 0.55). Beta diversity was assessed using a PLSDA plot, which revealed four clearly separated clusters (Figure 2C). ANOSIM analysis further established significant differences between all offspring groups (All *p* < 0.01).

**Figure 3:**
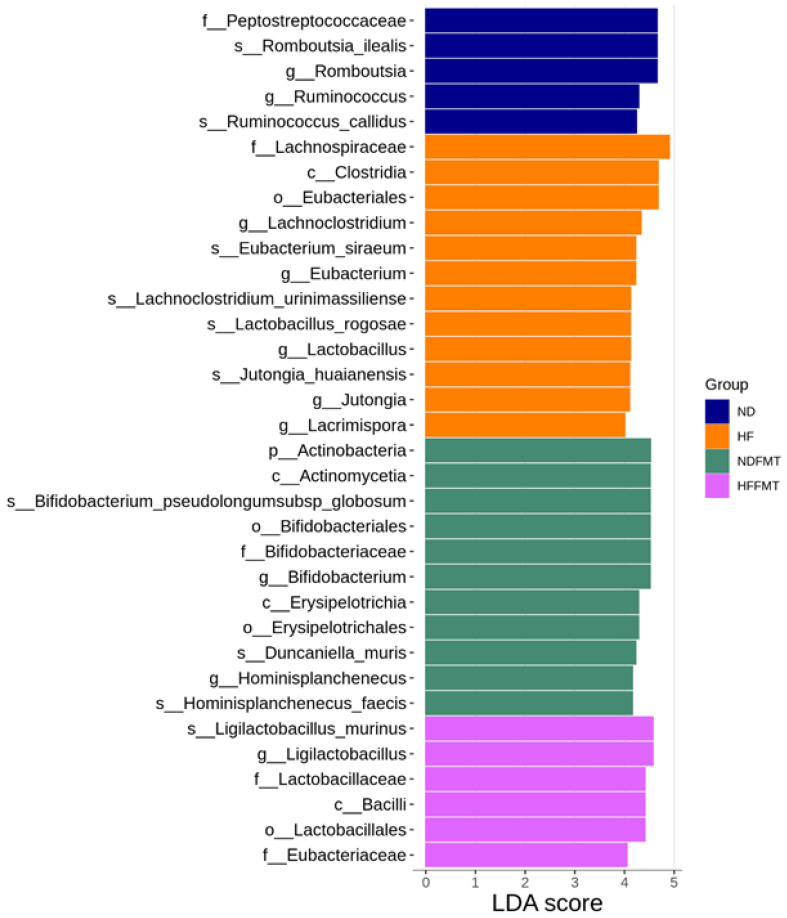
Linear discriminant analysis effect size (LEfSe) detects taxa exhibiting significant differential abundance among four offspring groups. Taxa with an LDA score exceeding 4 were emphasized as key contributors to group differences. N = 8/group. Statistical analysis by the Wilcoxon test.

Taxa showing the most pronounced differences in relative abundance across the experimental groups were identified using LEfSe analysis. Figure 3 revealed several bacterial genera and their corresponding higher taxonomic ranks were significantly enriched in specific groups. In the HF group, the genus *Lachnoclostridium*, along with its family, order, and class, displayed notably higher abundance. Other genera, including *Eubacterium, Lactobacillus, Jutongia*, and *Lacrimispora*, also showed elevated levels in the HF group, with LDA scores exceeding 4, indicating strong discriminative power. In contrast, the NDFMT group was characterized by a marked increase in *Bifidobacterium pseudolongum* and *Hominisplanchenecus faecis*, with enrichment extending across their respective genus, family, class, order, and phylum levels, highlighting the influence of maternal FMT from normal-diet donors on shaping offspring microbiota composition. Additionally, the HFFMT group displayed a significant enrichment of *Ligilactobacillus murinus* along with its associated taxonomic ranks, including genus, family, class, and order, suggesting that maternal FMT from HF-fed donors selectively modulates specific microbial populations in offspring (Figure 3).

**Figure 4:**
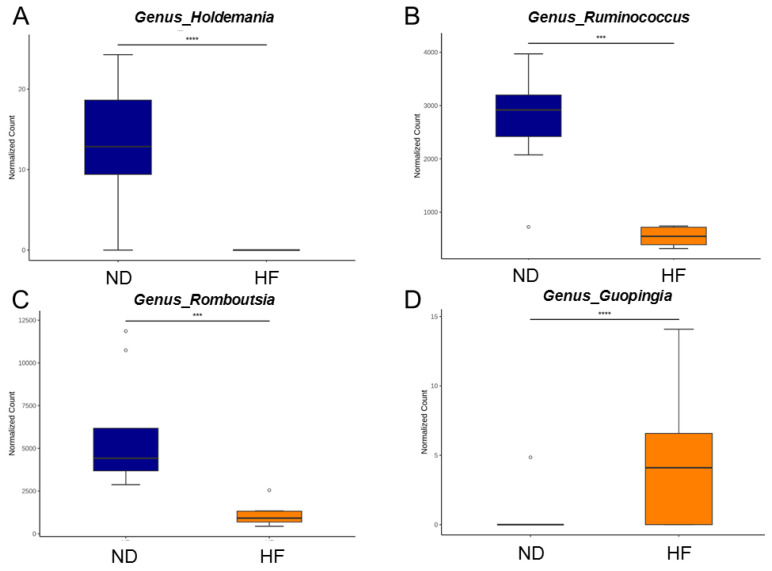
Relative abundance of the genera (**A**) *Holdemania*, (**B**) *Ruminococcus*, (**C**) *Romboutsia*, and (**D**) *Guopingia* between the ND and HF group. N = 8/group. Statistical analysis by using *p*-values adjusted for multiple comparisons with the false discovery rate (FDR) method. *** *p* < 0.005; **** *p* < 0.001.

Beyond these LEfSe-identified taxa, the maternal HF diet was associated with broader shifts in gut microbiota composition. Specifically, the relative abundance of genera such as *Holdemania, Ruminococcus,* and *Romboutsia* was reduced, whereas the genus *Guopingia* was increased in the HF-exposed offspring compared with controls (Figure 4).

**Figure 5:**
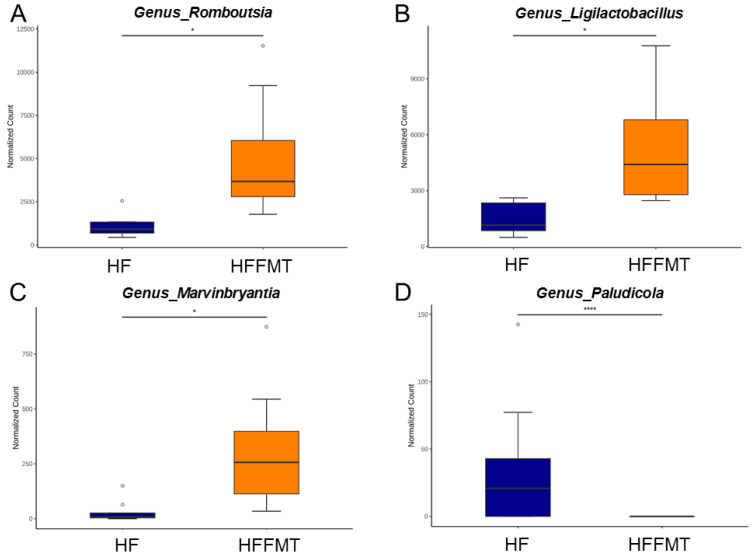
Relative abundance of the genera (**A**) *Romboutsia*, (**B**) *Ligilactobacillus*, (**C**) *Marvinbryantia*, and (**D**) *Paludicola* between the HF and HFFMT group. N = 8/group. Statistical analysis by using *p*-values adjusted for multiple comparisons with the false discovery rate (FDR) method. * *p* < 0.05; **** *p* < 0.001.

Compared to the HF group, FMT from donors consuming a normal diet led to significant increases in the genera R*omboutsia, Ligilactobacillus,* and *Marvinbryantia*, and a decrease in the genus *Paludicola* (Figure 5).

### 3.3. Short-Chain Fatty Acids and Their Receptors

**Figure 6:**
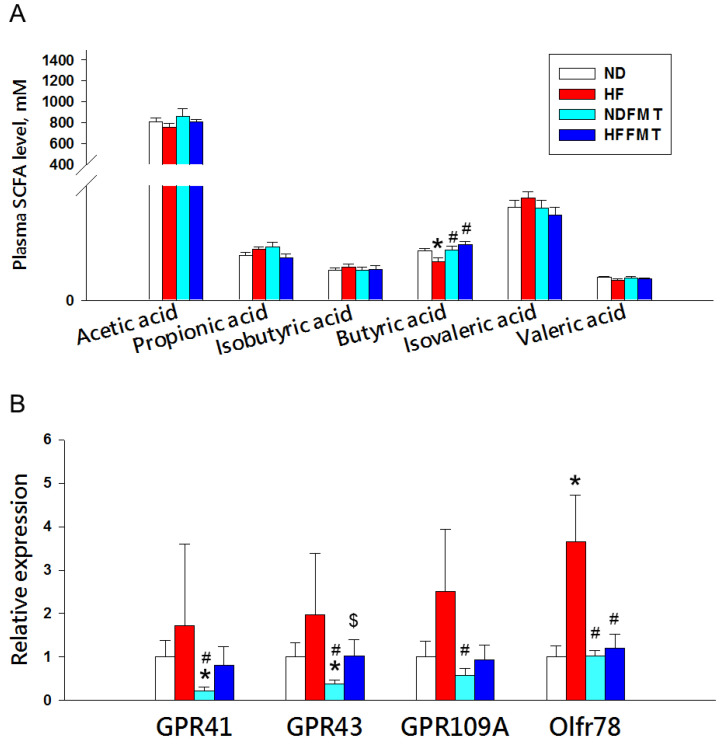
(**A**) Plasma concentrations of short-chain fatty acids (SCFAs) and (**B**) renal mRNA expression of SCFA receptors at week 12. N = 8/group. Statistical analysis by a one-way ANOVA followed by Tukey’s post hoc test. * *p* < 0.05 vs. ND; # *p* < 0.05 vs. HF; $ *p* < 0.05 vs. NDFMT.

Given the pivotal role of SCFAs in BP regulation, we measured their plasma levels and examined the expression of related receptors in kidney tissue (Figure 6). Offspring of dams fed a HF diet showed significantly reduced plasma butyrate concentrations (ND: 6.45 ± 0.30 mM vs. HF: 5.06 ± 0.45 mM, *p* < 0.05), which were restored following maternal FMT from ND donors (HFFMT: 7.23 ± 0.42 mM vs. HF: 5.06 ± 0.45 mM, *p* < 0.05) (Figure 6A). No significant changes were observed in plasma levels of acetate, propionate, isobutyrate, isovalerate, or valerate across groups. Kidney expression of the SCFA receptors GPR41 and GPR43 were diminished in the NDFMT group relative to both ND and HF groups. Conversely, HF exposure upregulated renal Olfr78 expression (ND: 1 ± 0.26-fold change vs. HF: 3.65 ± 1.07-fold change, *p* < 0.05), an effect reversed by FMT from ND donors in the HFFMT group (HFFMT: 1.2 ± 0.32-fold change vs. HF: 3.65 ± 1.07-fold change, *p* < 0.05) (Figure 6B).

### 3.4. Oxidative Stress

**Figure 7:**
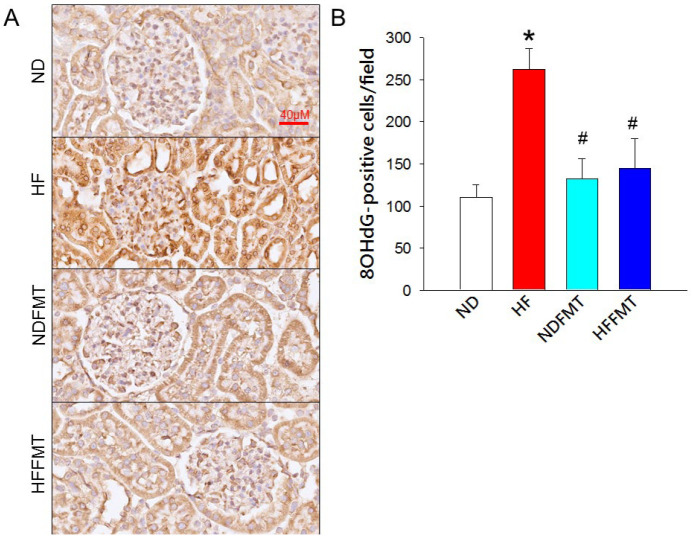
(**A**) Immunohistochemistry for 8-hydroxy-2′-deoxyguanosine (8-OHdG) showed predominant nuclear staining in both glomerular and tubular compartments of offspring kidneys, indicating nuclear DNA injury at these sites. (**B**) Quantification of 8-OHdG–positive nuclei within the renal cortex revealed group-dependent differences in oxidative DNA damage burden. Scale bar = 40 µM. N = 8/group. Statistical analysis by a one-way ANOVA followed by Tukey’s post hoc test. * *p* < 0.05 vs. ND; # *p* < 0.05 vs. HF.

We next investigated renal 8-OHdG immunoreactivity to explore the potential relationship between oxidative stress, maternal FMT intervention, and kidney injury induced by a maternal HF diet. As shown in Figure 7, offspring kidneys from the HF group exhibited predominant nuclear immunostaining in both glomerular and tubular epithelial cells, indicating extensive oxidative DNA damage. Although the representative low-magnification image may appear uniformly positive, semi-quantitative analysis revealed regional variation in staining intensity between glomerular and tubular compartments. Quantitative assessment further revealed a substantial increase in the amount of 8-OHdG-positive cells in these offspring kidneys compared with all other experimental groups, underscoring the impact of maternal HF consumption on offspring renal oxidative stress. In contrast, offspring from dams that received FMT from ND donors displayed markedly reduced 8-OHdG immunoreactivity, suggesting that maternal FMT intervention can effectively attenuate HF-induced oxidative injury in the kidney.

## 4. Discussion

This study is the first to demonstrate that maternal FMT from healthy donors mitigates offspring hypertension induced by maternal HF intake, likely through modulation of the gut microbiota, increased butyric acid production, downregulation of renal Olfr78 expression, and attenuation of oxidative stress. In contrast, maternal FMT from HF diet-fed donors into healthy dams promoted hypertension in offspring, further supporting the causal role of gut microbiota in mediating the adverse programmed effects of maternal HF diets.

Our key findings are as follows: (1) Maternal HF intake led to elevated BP in adult offspring, which was significantly attenuated by maternal FMT from healthy donors; (2) Offspring hypertension was associated with decreased plasma butyric acid levels, upregulation of renal Olfr78, and increased oxidative stress as reflected by elevated renal 8-OHdG staining; (3) Maternal HF diet and FMT intervention shaped distinct gut microbiota configurations in the offspring, forming four discrete enterotypes; (4) Maternal FMT from HF-fed donors promoted offspring hypertension, accompanied by downregulation of renal GPR41 and GPR43 expression—key receptors involved in SCFA signaling; and (5) The protective effects of FMT from healthy donors were linked to increased butyric acid levels, reduced Olfr78 expression, enrichment of beneficial taxa (e.g., Ligilactobacillus *murinus, Romboutsia*, and *Marvinbryantia*), and depletion of pro-hypertensive taxa such as *Paludicola*.

These findings provide novel evidence that maternal FMT represents a promising microbiota-targeted strategy to prevent hypertension of developmental origin. The observed BP elevations in our rodent model are within the range considered physiologically significant in preclinical studies of hypertension [9,10,11], reflecting sustained increases above control levels that are relevant for evaluating the impact of maternal fructose intake and FMT interventions. Previous research has linked maternal HF consumption to offspring hypertension [9,10,11], and several gut-directed interventions—including prebiotics, probiotics, and postbiotics—have shown partial protective effects [13,14]. Our data extend this body of work by demonstrating that FMT from healthy donors exerts antihypertensive effects, emphasizing the nutritional and therapeutic relevance of supporting maternal microbial stability, even if the resulting microbial community does not exactly replicate that of ND controls.

The mechanistic role of gut microbiota was further supported by significant shifts in microbial β-diversity and enrichment of butyrate-producing bacteria in response to FMT from healthy donors. Increased abundance of *Ruminococcus, Romboutsia,* and *Marvinbryantia* was associated with elevated plasma butyric acid, a key SCFA with vasoregulatory properties [22,23]. Butyrate has been shown to lower BP via inhibition of oxidative stress and modulation of SCFA receptors such as Olfr78 and GPR41 [24,25,26]. Consistent with this, offspring of HF-fed dams displayed renal upregulation of Olfr78, a receptor that promotes hypertension through renin release [27]. This response was normalized by FMT from healthy donors, restoring balanced SCFA signaling. In contrast, vasodilatory pathways mediated by GPR43 and GPR109A [28] were suppressed when maternal FMT derived from HF-fed donors was introduced, leading to downregulation of these receptors and a shift toward vasoconstriction with elevated BP. Collectively, these results support the existence of a microbiota–SCFA–host receptor axis that underlies the observed BP-lowering effects.

Notably, FMT also modulated specific taxa previously linked to hypertension. For example, *Ligilactobacillus murinus*—enriched by FMT—has been reported to reduce BP in spontaneously hypertensive rats [29], while *Ruminococcus* levels were normalized following FMT, consistent with a protective microbial signature [30]. While FMT from healthy donors conferred physiological benefits, the microbial community in HF rats did not fully resemble that of ND controls. This may reflect partial engraftment, host-specific influences, or the persistence of HF-associated microbiota, suggesting that additional strategies may be needed to achieve a more complete microbial shift.

We also confirmed a mechanistic role for oxidative stress [31,32,33], as maternal HF exposure increased kidney 8-OHdG expression—an established marker of oxidative DNA damage and renal injury. This response was significantly attenuated by maternal FMT from healthy donors, further supporting the antioxidant potential of microbial-derived butyrate and gut-targeted strategies.

FMT has emerged as a promising approach to restore gut microbial balance. In this study, we demonstrated that maternal FMT from healthy donors could mitigate offspring hypertension induced by maternal HF diets. These findings extend our previous work linking maternal diet, gut microbiota, and offspring BP, and highlight the potential of microbiota-targeted interventions. Nevertheless, several translational considerations should be noted. While rodent models allow mechanistic exploration, the human microbiota is more complex and shaped by diverse dietary and environmental factors [34]. Developmental timing also differs between species [35], and clinical FMT in humans requires rigorous donor selection and safety evaluation. In addition, mechanistic uncertainties remain regarding how specific microbial taxa and their metabolites interact with host signaling pathways to influence kidney programming. Sex differences represent another important dimension. Our study focused on male offspring to reduce variability, yet accumulating evidence suggests that females may exhibit distinct responses to both maternal diet and microbiota-targeted therapies [36]. Hormonal and metabolic factors likely contribute to these divergent outcomes, underscoring the importance of future sex-specific analyses. Building on our findings, we hypothesize that maternal FMT protects offspring by enhancing beneficial metabolites such as SCFAs and reducing detrimental ones, thereby influencing kidney programming pathways. These effects may be sex-dependent. Longitudinal human studies will be essential to test these hypotheses and determine the feasibility and safety of maternal FMT as a preventive strategy in high-risk pregnancies.

Despite the novel insights provided by this study, several limitations should be carefully considered. First, we employed ex vivo FMT without prior antibiotic pretreatment or the use of germ-free models. Although this approach effectively produced physiological effects, engraftment efficiency may be limited. As a result, the transferred microbial community may not fully replicate the donor profile, potentially affecting the fidelity and reproducibility of the intervention. Furthermore, while our study emphasizes microbiota-mediated effects, we cannot exclude contributions from parallel mechanisms such as immune modulation and host metabolic adaptation. Future studies using antibiotic pretreatment or germ-free models could help optimize engraftment and strengthen the interpretation of FMT-mediated effects. Second, the rectal administration route for FMT bypasses the upper gastrointestinal tract, potentially limiting colonization dynamics and microbial interactions that normally occur during transit through the stomach and small intestine. Third, although we characterized microbial composition and measured SCFA concentrations, functional validation using metagenomic, transcriptomic, or metabolomic approaches is necessary to confirm mechanistic links and establish causality [37]. Fourth, all observations in this study remain correlative, and further mechanistic experiments, such as SCFA receptor knockout or knockdown models, are required to directly define causal pathways underlying the observed physiological effects. Finally, this study primarily focused on the kidney; however, the beneficial effects of FMT may also involve other organs that regulate BP, including the heart, brain, and vasculature, which warrant further investigation.

## 5. Conclusions

In summary, this study demonstrates that maternal FMT from healthy donors protects offspring against hypertension induced by maternal HF intake, through modulation of the gut microbiota, SCFA production, host receptor signaling, and oxidative stress. These results highlight the nutritional significance of maternal microbial environments in shaping offspring cardiometabolic health. However, key limitations—including the ex vivo FMT model, focus on male offspring, and uncertain human translatability—underscore the need for mechanistic clarification and longitudinal clinical studies before maternal FMT can be advanced as a preventive strategy.

## Figures and Tables

**Table 1:** Weights and functional parameters in male offspring at 12 weeks of age.

Group	ND	HF	NDFMT	HFFMT
Body Weight, g	392 ± 9	426 ± 8	447 ± 12 *	421 ± 8
Left Kidney Weight (KW), g	1.77 ± 0.08	1.95 ± 0.04	1.85 ± 0.06	1.74 ± 0.06
Left KW/100 g BW	0.45 ± 0.02	0.46 ± 0.01	0.42 ± 0.01 #	0.41 ± 0.01 #
Systolic blood pressure, mmHg	131 ± 2	144 ± 1 *	141 ± 1 *	136 ± 1 *#$
Diastolic blood pressure, mmHg	85 ± 1	95 ± 2 *	93 ± 3	87 ± 3
Mean Arterial Pressure, mmHg	101 ± 1	111 ± 1 *	109 ± 2 *	103 ± 2 #
24 h creatinine clearance, mL/min/kg body weight	4.49 ± 0.17	4.36 ± 0.81	4.5 ± 0.88	3.52 ± 0.76

N = 8/group. Statistical analysis by a one-way ANOVA followed by Tukey’s post hoc test. * *p* < 0.05 vs. ND; # *p* < 0.05 vs. HF; $ *p* < 0.05 vs. NDFMT.

## Data Availability

Data are contained within the article.
